# Uptake and intracytoplasmic storage of pigmented particles by human CD34+ stromal cells/telocytes: endocytic property of telocytes

**DOI:** 10.1111/jcmm.12437

**Published:** 2014-09-30

**Authors:** Lucio Díaz-Flores, Ricardo Gutiérrez, Mª Pino García, Francisco J Sáez, Fernando Aparicio, Lucio Díaz-Flores, Juan F Madrid

**Affiliations:** a Department of Anatomy, Pathology, Histology and Radiology, Faculty of Medicine, University of La LagunaTenerife, Spain; b Department of Cell Biology and Histology UFI11/44, School of Medicine and Dentistry, University of the Basque Country, UPV/EHULeioa, Spain; c Department of Cell Biology and Histology, School of Medicine, Regional Campus of International Excellence. “Campus Mare Nostrum”, University of MurciaEspinardo, Spain

**Keywords:** CD34+ stromal cells, telocytes, endocytosis, phagocytosis, colon, skin

## Abstract

We studied the phagocytic-like capacity of human CD34+ stromal cells/telocytes (TCs). For this, we examined segments of the colon after injection of India ink to help surgeons localize lesions identified at endoscopy. Our results demonstrate that CD34+ TCs have endocytic properties (phagocytic-like TCs: phTCs), with the capacity to uptake and store India ink particles. phTCs conserve the characteristics of TCs (long, thin, bipolar or multipolar, moniliform cytoplasmic processes/telopodes, with linear distribution of the pigment) and maintain their typical distribution. Likewise, they are easily distinguished from pigment-loaded macrophages (CD68+ macrophages, with oval morphology and coarse granules of pigment clustered in their cytoplasm). A few c-kit/CD117+ interstitial cells of Cajal also incorporate pigment and may conserve the phagocytic-like property of their probable TC precursors. CD34+ stromal cells in other locations (skin and periodontal tissues) also have the phagocytic-like capacity to uptake and store pigments (hemosiderin, some components of dental amalgam and melanin). This suggests a function of TCs in general, which may be related to the transfer of macromolecules in these cells. Our ultrastructural observation of melanin-storing stromal cells with characteristics of TCs (telopodes with dichotomous branching pattern) favours this possibility. In conclusion, intestinal TCs have a phagocytic-like property, a function that may be generalized to TCs in other locations. This function (the ability to internalize small particles), together with the capacity of these cells to release extracellular vesicles with macromolecules, could close the cellular bidirectional cooperative circle of informative exchange and intercellular interactions.

## Introduction

Telocytes (TCs) are a newly described distinct type of stromal cell [1–3], located in multiple anatomical sites in both perivascular and stromal positions in the connective tissue. They are mainly distinguished from other types of cells by their distribution (location), relationship, morphology, immunohistochemical profile and ultrastructural characteristics. A specific immunohistochemical marker for TCs has not been found [2]. Nevertheless, expression of CD34 in stromal cells is the best available immunohistochemical choice for TC identification [2]. PDGFRβ and PDGFRα are also specific for TCs [4,5]. Detection is more precise when previous studies have confirmed that CD34+ stromal cells are TCs in a specific tissue or organ, as occurs in the enteric wall [2,5–7].

Several roles for TCs have been postulated, including the following: (*i*) paracrine and/or juxtacrine intercellular modulation (cell-to-cell signalling by either intercellular junctions or extracellular vesicles – multivesicular bodies – or shed microvesicles or exosomes [8,9], (*ii*) neurotransmission (*e.g*. contributing to spread the slow waves generated by interstitial cells of Cajal (ICCs) and participation in the control of muscle tone and motor activity [2,5,9], (*iii*) mechanical support (TCs as resistant and deformable cells following stretches [2,6,10,11], (*iv*) migration guide for other cells during development, renewal and repair [11,12], (*v*) modulation of local homoeostasis, organization of the extracellular matrix and integration of neural or vascular input with the organ function [13], (*vi*) control and regulation of other cell types, mainly of stem cells (control of growth and differentiation) in stem cell niches (TCs and stem cells make a tandem: TCs as the ‘nurse’ of progenitor cells in stem cell niches) [14–17], (*vii*) immunomodulation and immunosurveillance [18] and (*viii*) in neoangiogenesis (physical contacts and chemical signalling, *e.g*. VEGF and nitric oxide) [13,19,20]. In a preliminary study, our group suggested a certain capacity of stromal cells for the uptake and intracellular accumulation of particles (endocytic/phagocytic-like property/macrophage-like capacity) [21]. It is therefore of interest to assess this possible phagocytic-like capacity in the enteric wall, in which CD34+ stromal cells are TCs [2,5–7].

Given the above, this study was undertaken to assess the phagocytic-like property (capacity of uptake and storage of India ink particles) of tissue-resident TCs in the segments of the colon after injection of India ink to help surgeons localize lesions identified at endoscopy. Our results revealed that India ink particles were uptaken (endocytozed) and stored by TCs (phagocytic-like TCs: phTCs) and macrophages. Consequently, new studies were planned to investigate: (*i*) whether other non-TC and non-macrophage cells in the enteric wall (mainly interstitial cell of Cajal: ICC) have this property, (*ii*) whether TCs show this uptake activity in other locations after interstitial release of endogenous or exogenous pigmented particles (hemosiderin, silver component of amalgam and melanin) and (*iii*) the differences between phTCs and CD68+ specialized phagocytic cells (CD68+ macrophages) after phagocytosis.

## Material and methods

### Tissue samples

Specimens were obtained and selected from the archives of the Department of Anatomy, Histology, Pathology and Radiology of the Faculty of Medicine/University Hospital of the Canary Islands, and the Department of Pathology, Hospiten® Hospitals. To assess the capacity of intestinal TCs to uptake and store India ink particles, specimens were selected from unaffected areas of 12 cases of surgical resected segments of colonic tattooing with India ink for the preoperative marking of colonic zones with tumours, including tubular and villous adenomas. Extraintestinal-stained adipose tissue (black maculae) and lymph nodes were also obtained in 3 and 2 cases, respectively. To investigate the uptake activity of TCs in other locations, the following specimens were selected from: (*i*) four cases of pigmented purpuric dermatoses with hemosiderin deposition, (*ii*) three cases of oral amalgam pigmentation in periodontal tissues (formerly known as localized argyria) and (*iii*) eight cases of compound pigmented/melanocytic nevi. Cases of pigmented melanocytic nevi (n: 2) had also been processed for electron microscopy. Normal tissues were also obtained from surgical specimens (borders free of lesion), and similar characteristics of CD34+ stromal cells/TCs to those previously described by other authors were observed [2,5,6,20]. Moreover, the phagocytic-like cells conserve the morphology and distribution of CD34+ stromal cells/TCs (see Results of phagocytic-like cells with endocytozed particles). All protocols were performed in accordance with international ethical guidelines.

### Light microscopy

Specimens were fixed in a buffered neutral 4% formaldehyde solution, embedded in paraffin and cut into 4 μm-thick sections, which were stained with haematoxylin and eosin, and Perl Prussian blue stain for iron (in cases of pigmented purpuric dematoses).

### Immunohistochemistry

Three-μm-thick sections were cut and attached to silanized slides. After pre-treatment for enhancement of labelling [antigen retrieval PT-Link (Dako, Glostrup, Denmark), Ref. 1012], the sections were blocked with 3% hydrogen peroxide and then incubated with primary antibodies (Dako; 10–40 min.). The primary antibodies used in this study were as follows: CD34 (1:50), code no. IR63261; CD-31 (dilution 1:50), code no. IR61061; alpha smooth muscle actin (αSMA; dilution 1:50), code no. IR61161; CD68 (dilution 1:50), code no. IR 60961; CD117 (c-kit; dilution 1:50), code no. A-4502; vimentin (dilution 1:100), code no. IR 630; and h-caldesmon (dilution 1:50), code no. IR05461. The immunoreaction was developed in a solution of diaminobenzidine and the sections were then briefly counterstained with haematoxylin, dehydrated in ethanol series, cleared in xylene and mounted in Eukitt®, O. Kindler GmbH, Freiburg, Germany. Positive and negative controls were used.

### Transmission electron microscopy

For electron microscopy, small pieces were fixed in a glutaraldehyde solution, diluted to 2% with sodium cacodylate buffer, pH 7.4, for 6 hrs at 4°C, washed in the same buffer, post-fixed for 2 hrs in 1% osmium tetroxide, dehydrated in a graded ethanol series and embedded in epoxy resin. Semithin sections (1.5 μm) were mounted on acid-cleaned slides and stained with 1% Toluidine blue. Ultrathin sections were double-stained with uranyl acetate and lead citrate. The grids were examined at 60 kV with a JEOL, Jeol Ltd. Tokyo, Japan 100B electron microscope.

## Results

### Phagocytic-like TCs with endocytozed particles in the colonic segment tattooed with India ink

**Fig. 1:**
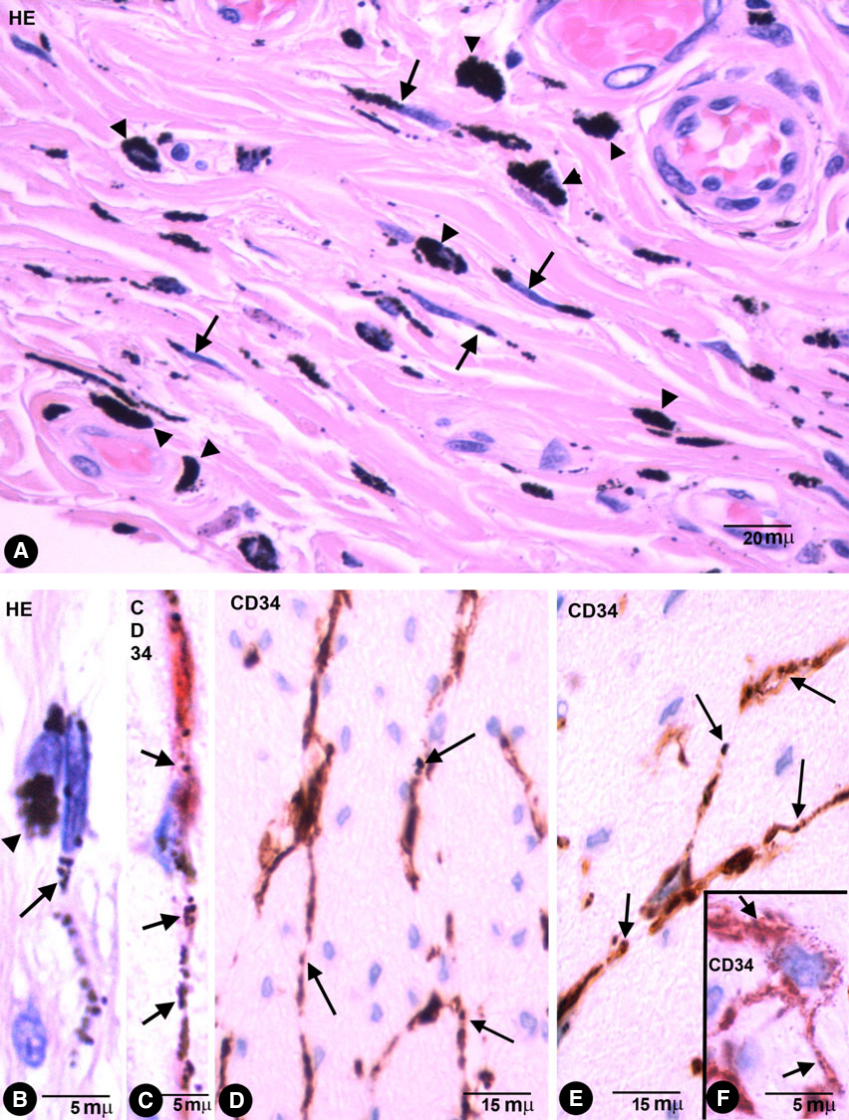
Stromal cells in enteric wall tattooed with India ink. (**A**) Characteristics of stromal cells (arrows) and macrophages (arrow-heads) in the submucosa with engulfed pigmented particles in an haematoxylin and eosin stained section. Note that the intracellular pigment in some stromal cells shows a linear distribution drawing their processes. (**B**) Detail of a stromal cell (arrow) and a macrophage (arrowhead) with endocytozed particles. (**C**) A bipolar CD34+ phTC with intracellular pigment (arrows) in submucosa. (**D**–**F**) bipolar and Multipolar CD34+ phTCs with intracellular pigment (arrows) between SMCs of muscular propria (**D** and **E**) and myenteric plexus ganglia (**F**).

**Fig. 2:**
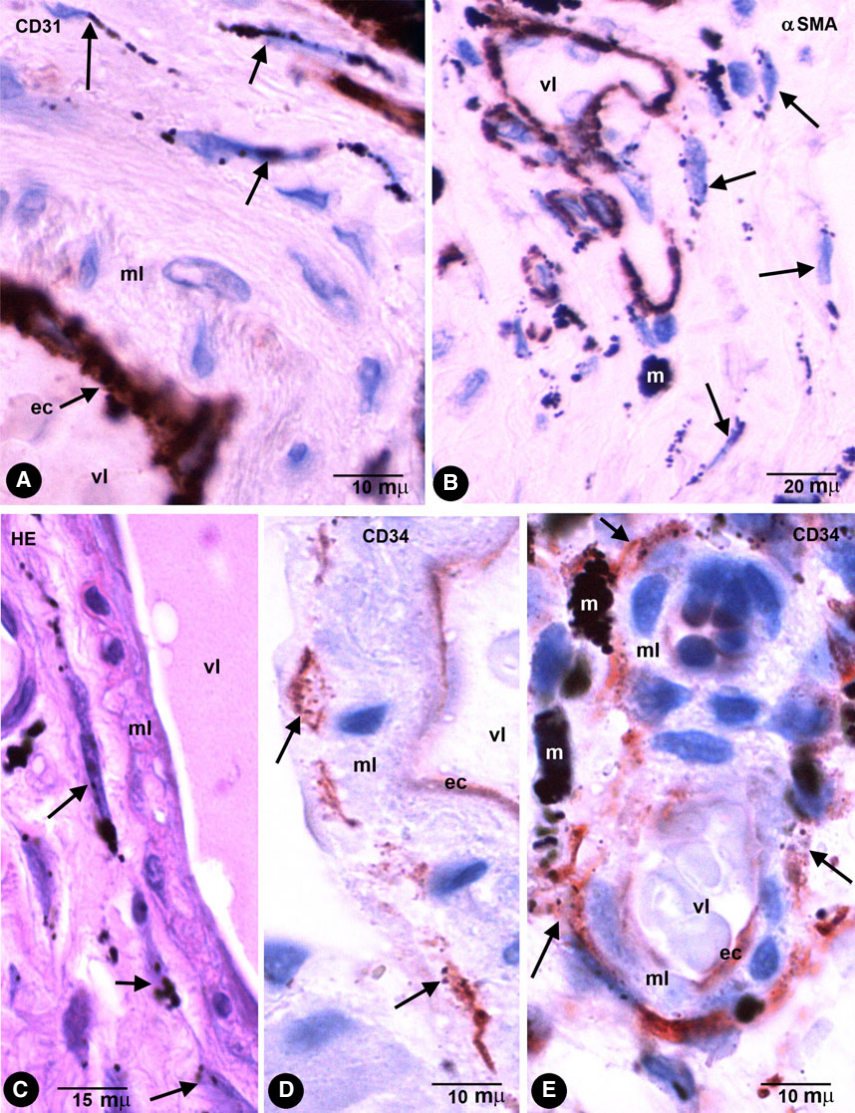
Stromal cells in intestinal wall tattooed with India ink. (**A** and **B**) The stromal cells containing endocytozed particles (arrows) are negative for CD31 (**A**) and for αSMA (**B**). Endothelial cells are stained by CD31 (**A**) and cells in the vessel media layer by anti-αSMA (**B**). (**C**) Stromal cells with intracellular pigment (arrows) around a vessel in an haematoxylin and eosin stained section. (**D** and **E**) CD34+ phTCs with intracytoplasmic India ink particles (arrows) around different-sized vessels. vl, Vessel lumen; ec, Endothelial cell; ml, Medial layer; m, Macrophage.

Stromal cells and macrophages with intracytoplasmic-pigmented particles were seen in the specimens of the colonic segments tattooed *in vivo* with India ink (Fig. 1). Some extracellular particles of pigment were also present in the interstitium. The stromal cells with pigmented particles expressed CD34 (CD34+ phTCs) and where the pigment was released practically all the CD34+ TCs showed pigment. CD34+ phTCs displayed a small oval or triangular body and long, thin bipolar or multipolar cytoplasmic processes (telopodes; Fig. 1). The intracellular pigment was present in the somatic region around the nucleus and in the telopodes. phTCs were also stained with anti-vimentin and did not express CD45, CD68, CD31 (Fig. 2A), αSMA (Fig. 2B), or h-caldesmon. In tissue sections stained with haematoxylin and eosin, the intracellular pigment showed a peculiar linear distribution (Figs 1A, B and 2C), drawing the processes of some stromal cells whose morphology and arrangement coincided with that of the anti-CD34 stained phTCs. (In Figs 1 and 2, compare stromal cells obtained from tissue sections stained with haematoxylin and eosin and with anti-CD34/CD34+ phTCs.)

**Fig. 3:**
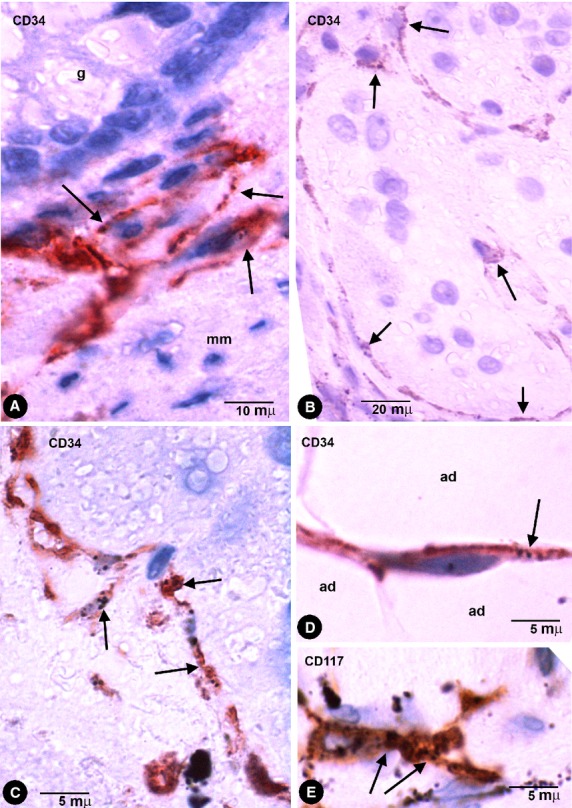
Enteric wall tattooed with India ink. (**A**) CD34+ phTCs around the basal portion of a gland (g) and lining the mucosa surface of muscularis mucosae (mm). (**B** and **C**) CD34+ phTCs (arrows) arranged around myenteric plexus ganglia. (**D**) A CD34+ phTC (arrow) between adipocytes (ad). (**E**) An occasional CD117 (c-kit) stained interstitial cell of Cajal (ICC) with endocytozed India ink particles (arrows).

CD34+ phTCs were observed in some deep regions of the mucosa and in the submucosa, muscular propria and serosa. In the rest of the mucosa, CD34+ TCs or CD34+ phTCs were not detected. In all pigmented layers of the enteric wall, phTCs surrounded vessels of different calibre (encircling the media layer; Fig. 2) and nerves. In the deep region of the lamina propria, scattered phTCs were located around the basal portion of some intestinal glands (Fig. 3A). In the muscularis mucosae, abundant phTCs were observed in its mucosa (Fig. 3A) and submucosa surfaces, and around groups of smooth muscle cells (SMCs). In the submucosa, phTCs formed networks between collagen and elastic fibres and two almost continuous layers bordering on the muscularis mucosae and the muscular propria covering the submucosal border of the circular muscle layer. The submucous plexus ganglia were also encircled by phTCs. In both layers of muscular propria, phTCs were arranged around fascicles and bundles of SMCs (in septal borders) and between SMCs (Fig. 1D and E). In Auerbach's plexuses, phTCs surrounded ganglia (covering cells; Fig. 3B and C) and formed networks in nerve strands and between the ganglia (Fig. 1F). CD34+ phTCs were also present in peri-intestinal adipose tissue when the injected pigment spread towards it. In this location, phTCs formed a delicate network that surrounded adipose lobes and lobules. The cytoplasmic bodies and telopodes of phTCs were also closely associated with adipocytes (Fig. 3D).

### Behaviour of other non-TCs and non-macrophage cells in colonic segments tattooed with India ink

Other cells in the stroma, including those of the vascular wall, did not show uptake or storage of India ink, except for a few c-kit/CD117-stained ICCs (Fig. 3E), when the pigment was directly released in the interstitium as occurred in our cases.

### CD34+ cells with a telocytic appearance and endocytozed pigmented particles in other locations

In all locations studied, in which different types of pigment were released in the interstitium, the pigment was incorporated into the cytoplasm of CD34+ cells and macrophages.

**Fig. 4:**
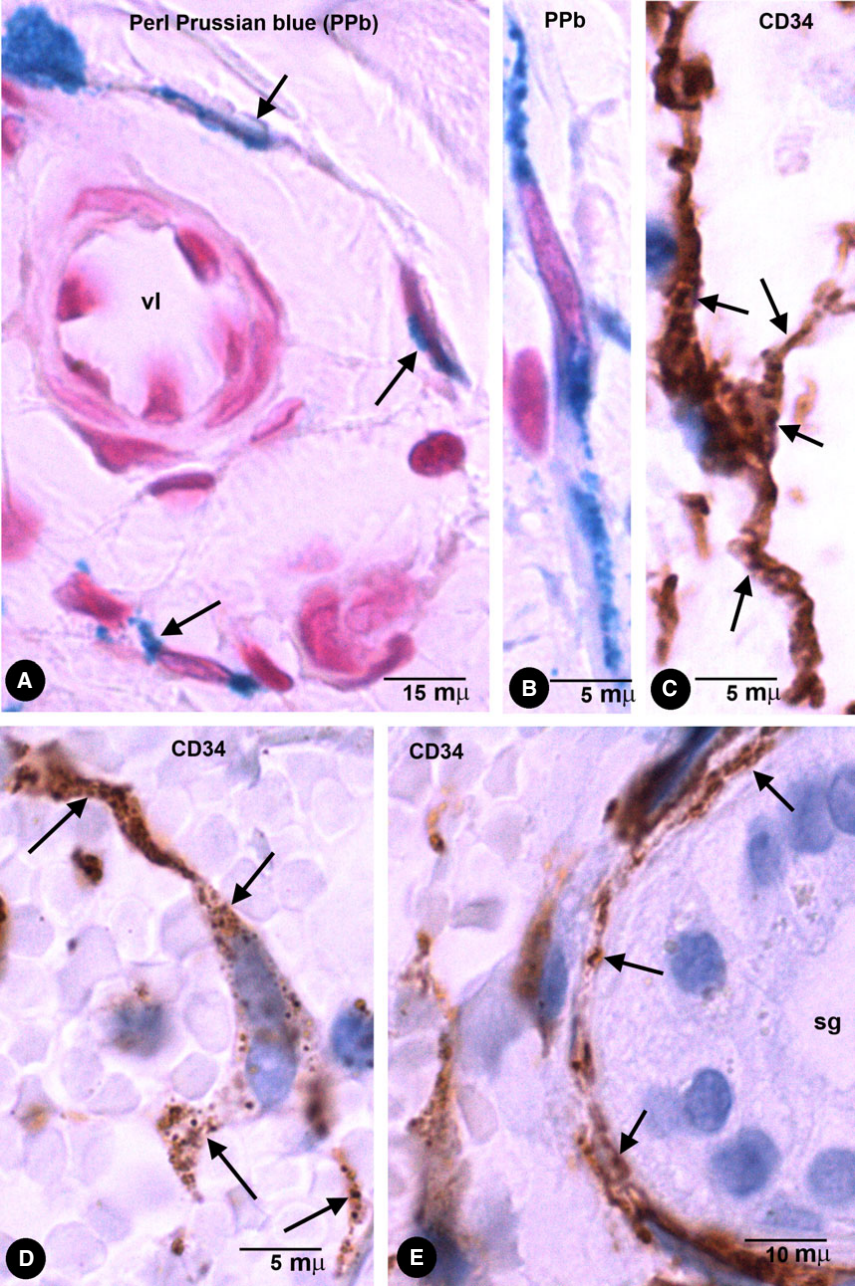
Stromal cells with intracytoplasmic pigmented particles in purpuric dermatoses (hemosiderin deposition). (**A** and **B**) Stromal cells, around a vessel (**A**) and in the interstitium (**B**) of the reticular dermis, obtained from sections with Perl Prussian blue stain. (**C**–**E**) CD34+ stromal cells with endocytozed particles of hemosiderin (arrows) in the reticular dermis (**C** and **D**) and around a sweat gland (**D**, sg).

In pigmented purpuric dermatoses with hemosiderin deposition, stromal cells with intracytoplasmic iron deposition (Perl Prussian blue stained) were present in the dermis (Fig. 4A and B). CD34+ cells with appearance of TCs and with pigmented intracytoplasmic particles were observed (Fig. 4C–E). These cells showed an arrangement around vessels and skin annexes, such as sweat glands (Fig. 4E).

**Fig. 5:**
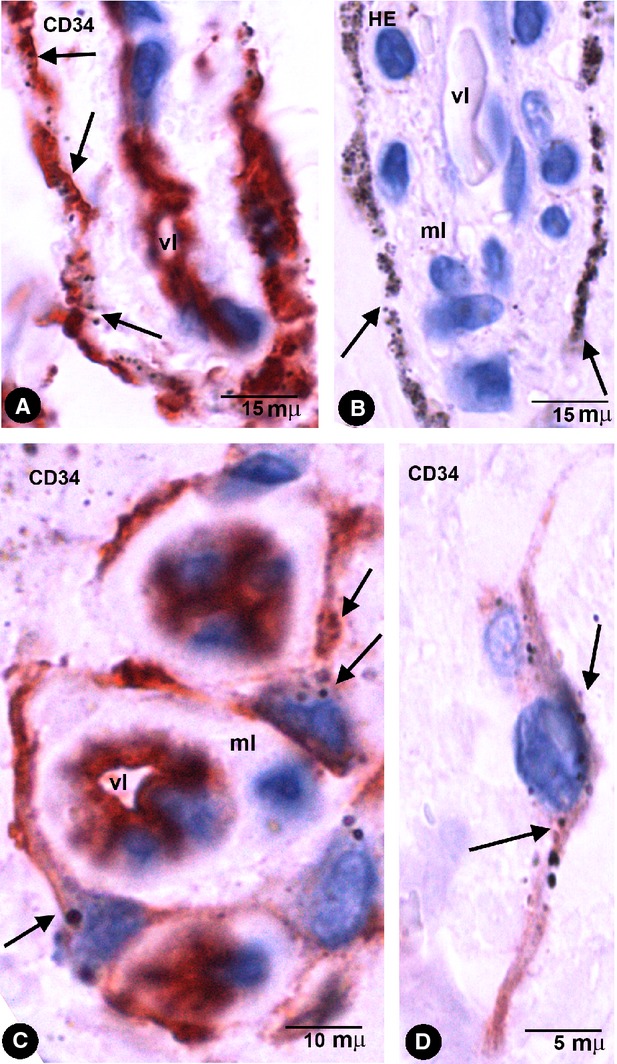
Stromal cells with intracytoplasmic pigmented particles in periodontal tissues with amalgam pigmentation. (**A** and **C**) CD34+ stromal cells with endocytozed particles (arrows). Note the arrangement of CD34+ stromal cells around different-sized vessels. (**B**) Stromal cells with abundant pigment around the media layer of a vessel in an HE-stained section. Note similar arrangement to CD34+ stromal cells in **A**. (**D**) A bipolar CD34+ stromal cell in the dermal interstitial compartment. Vl, Vessel lumen; ml, Media layer.

In amalgam pigmentation of the gingival mucosa (localized argyria), pigmented particles were stored in CD34+ cells with long, thin processes, arranged in a perivascular (Fig. 5A and C) and stromal distribution (Fig. 5D). A similar perivascular arrangement of the cells with pigment was observed in haematoxylin-stained sections (Fig. 5B). Foreign-body reaction and repair findings were associated in one case.

**Fig. 6:**
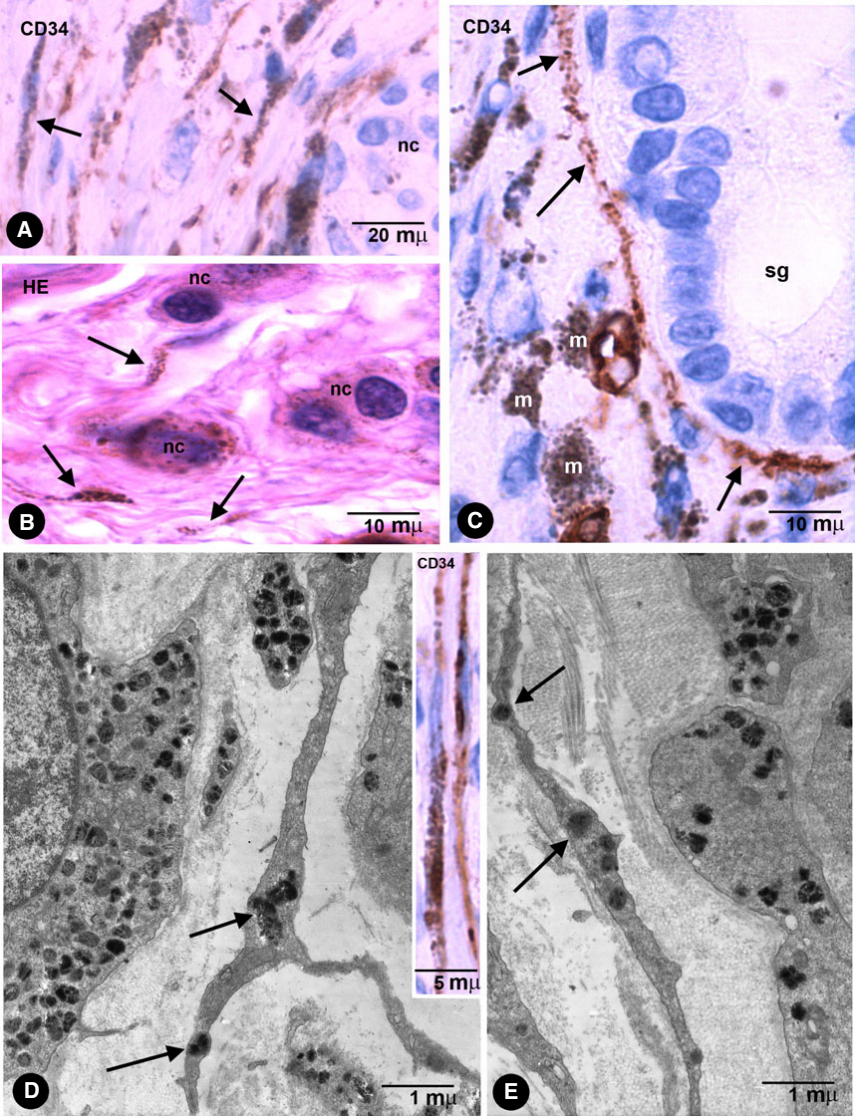
(**A** and **C**) CD34+ phTCs with pigment (arrows) near a nest of naevus cells (**A**, nc) and around a sweat gland (**C**, sg) in pigmented melanocytic nevi. (**B**) Stromal cells with pigment (arrows) near naevus cells (nc) in an haematoxylin and eosin stained section. (**D** and **E**) phTCs with ultrastructural characteristics of TCs show endocytozed melanosomes (arrows). Note that naevus cells present a basal lamina, whereas the TCs do not. In the insert of **D**, CD34+ phTCs with intracytoplasmic pigment. m, Macrophages.

In compound melanocytic nevi, pigmented intracytoplasmic particles were observed in CD34+ stromal cells arranged near nests of naevus cells (Fig. 6A) and skin annexes (Fig. 6C). In tissue sections stained with haematoxylin and eosin, stromal cells with intracellular pigment were also observed near naevus cells (Fig. 6B). Under electron microscopy, the particles were endocytozed melanosomes and the cells showed ultrastructural characteristics of TCs (long, thin telopodes with podomeres and podoms, dichotomous branching pattern and absence of basal membrane; Fig. 6D and E).

### General characteristics of macrophages after phagocytosis of pigmented particles

**Fig. 7:**
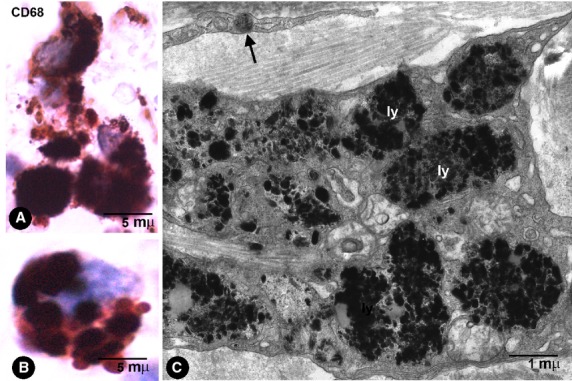
Macrophage characteristics. (**A** and **B**) CD68+ macrophages with abundant thick granules of India ink pigment in the intestinal wall. (**C**) Ultrastructural characteristics of macrophages in a pigmented melanocytic naevus. Note the abundant particles in lysosomes (ly). In the upper part of the image, a telopode of a phTC with an endocytozed melanosome (arrow).

Pigment was evident in macrophages, which were wandering in the stroma or accumulated near to vessels in the enteric wall and extraintestinal adipose tissue (Figs 1A and B, 2E, 6B and 7). In the lymph nodes, macrophages were observed in the intranodal sinuses. Macrophages displayed a rounded, oval or irregular morphology and abundant intracytoplasmic pigment. Furthermore, the pigment in macrophages appeared in thick granules or aggregates of granules, which occupied most of the cytoplasm. These cells expressed CD68 (Fig. 7A and B) and CD45 (which were only detected in the scarce cytoplasm unoccupied by the pigment), but were not stained by anti-CD31, anti-CD34, anti-αSMA or anti-h-caldesmon. In the enteric wall, macrophages were scarce in the muscular propria and plexuses, and more abundant in submucosa and serosa. Ultrastructurally (in pigmented melanocytic nevi), the cytoplasm of macrophages was packed with accumulated pigment particles (Fig. 7C). The particles appeared within membrane-limited lysosomes, whose shape, size and internal structure varied considerably. No transitional forms between phTCs and macrophages were observed.

## Discussion

Our observations demonstrate that tissue-resident CD34+ stromal cells, macrophages and scarce c-kit+ ICCs in the enteric wall (segments of the colon after injection of India ink) have the capacity to take up and store India ink particles. CD34+ stromal cells in the enteric wall are TCs, as previously described by other authors [2,5–7] and in our results practically all the CD34+ TCs showed pigment where it was released. Therefore, TCs are the principal non-macrophage cells with phagocytic-like properties in this location. Nevertheless, experimental studies using fluorescent particles could totally exclude the co-localization of CD34 and c-kit in the few cells expressing c-kit (ICCs) with the capacity for particle uptake. After endocytosis, CD34+ phTCs conserve characteristics of TCs, such as a slender nuclear body and long, thin varicose processes (telopodes). Furthermore, CD34+ phTCs maintain the typical distribution of TCs described in the literature in the layers of the enteric wall (deep region of lamina propria, muscularis mucosae, submucosa, muscular propria and serosa), mainly around blood vessels, muscle fascicles, nerves and intestinal plexuses [2,5–7].

Other non-phTCs and non-macrophages cells in the stroma (including those of the vascular wall) did not show uptake or storage of India ink, except a few ICCs. However, since the pigment was directly released into the interstitium, it is possible that other results could have been obtained in the vessel wall if the particles had been injected intravascularly. It has been reported that cells expressing *in vitro* CD34 at a high level and c-kit at a very low level differentiate into ICCs after loss of CD34 expression and increase in c-kit expression [22]. In this order, TCs have been considered as a pool of ICC precursors that renew ICCs undergoing apoptosis [2,6,23]. Therefore, it is possible that some ICCs may conserve the phagocytic-like property of their precursors.

Our study of CD34+ stromal cells in other locations (skin and periodontal tissues), after interstitial release of endogenous or exogenous pigmented particles, demonstrates that these cells can also take up and store the pigment (hemosiderin, melanin and some components of dental amalgam). In these locations, the characteristics of CD34+ stromal cells with intracytoplasmic pigment strongly suggest that they are also TCs and that this phagocytic-like capacity can be considered as another function of TCs in general. Our ultrastructural observation of melanin-storing stromal cells with TC characteristics in melanocytic nevi favours this possibility.

Former experimental studies, in which India ink was injected in the dermis and subcutis of mice, demonstrated the presence of endocytozed particles of pigment in cells described by the authors as macrophages and fibroblasts [24]. The authors considered that the uptake of indigestible and non-toxic material for fibroblasts protected the skin from injury or harm in the absence of an inflammatory response (non-inflammatory defence mechanism) [24]. A similar description was given for amalgam tattoos [25], in which non-macrophage cells with silver intracytoplasmic particles were also considered as fibroblasts [25]. Our results demonstrate that the fibroblast-like cells are CD34+ cells. An important finding is that these fibroblast-like cells remain in the tissue with unchanging forms and arrangement, enabling *in situ* pigment persistence. In one case of amalgam tattoos, granulomas around metallic material and repair features were present, inducing activation of the CD34+ stromal cells, which suggests that these cells may transform into fibroblasts and myofibroblasts [21].

The distinction between phTCs and macrophages is obvious. Indeed, in the enteric wall, phTCs are easily distinguishable from macrophages by their morphological and immunohistochemical characteristics. This distinction is possible even in sections stained with haematoxylin and eosin or non-specific markers, since macrophages are rounded, oval or irregular, with coarse granules of pigment clustered in their cytoplasms. Conversely, in phTCs, there are fewer intracytoplasmic particles, whose peculiar linear distribution along the cell processes (‘drawing’ the telopodes) allows a glimpse of their morphology. Furthermore, as previously mentioned, phTCs maintain their typical distribution, whereas macrophages appear wandering in the stroma or accumulated near vessels in the enteric wall and migrate towards lymph nodes. Likewise, phTCs are anti-CD34+ and anti-CD68-, while macrophages are anti-CD34- and anti-CD68+. Under electron microscopy (in skin with melanocytic pigmented nevi), the differences are even more evident.

In addition to a protective action in the absence of an inflammatory response, the uptake of small particles by TCs could be related to the transfer of macromolecules in these cells. Indeed, the release of extracellular vesicles (multivesicular bodies or shed microvesicles or exosomes) has been widely described [8,9,11,13]. Macromolecules, proteins, various RNAs and miRs in these extracellular vesicles could be endocytozed by neighbouring TCs or ICCs. Therefore, the phagocytic-like property described herein, together with the capacity of these cells to release extracellular vesicles with macromolecules, could close the cellular bidirectional cooperative circle of informative exchange and of intercellular interactions involved in modulating local homoeostasis.

The presence of phTCs lining the bases of intestinal crypts concurs with the tandem arrangement of TCs-adult stem cells in subepithelial niches described in several locations [8,14,15]. Indeed, crypt base columnar cells generate all epithelial lineages and represent the stem cells of the small intestine and the descending colon [26–28]. Popescu *et al*. point out that the distribution and interactions of TCs suggest that they are members of stem cell niches, playing the role of ‘nurse’ cells for adjacent progenitor/stem cells [8,14–16]. Thus, progenitor/stem cells will proliferate and differentiate only after signals from TCs [14,15,20,29]. In addition, several observations have also suggested a mesenchymal capacity for a subset of resident quiescent slow-cycling stromal cells with high proliferative potential, which express CD34 and which may be TCs, because of their general characteristics [21,30,31]. Likewise, PDGFRα and PDGFRβ are expressed in TCs and mesenchymal stem cells, a similarity that also supports the hypothesis that TCs represent adult stromal mesenchymal cells [4–6]. Therefore, TCs may not only be the *nurse* of progenitor cells but could also have a progenitor capacity *per se* (mesenchymal cell capacity). This probable role broadens the scope of this new type of cell, described and widely studied by Popescu *et al*., mainly in repair and regenerative medicine. In this way, TC capacity to ingest and store endogenous and exogenous particles demonstrated in this work also concurs with the probable mesenchymal capacity of TCs. Indeed, the ability of progenitor/stem cells to phagocytoze is supported by various findings, such as several macrophage characteristics of stem cells [32–34], isolation of pluripotent stem cells by means of macrophage surface antigens [35–37], and presence of CD34 expression and mesenchymal multipotency in an adipose resident population with phagocytic properties [38]. Whether TCs represent adult stromal mesenchymal stem cells and whether endocytic activity is present in activated TCs and descendent cells need further studies.

In conclusion, our results indicate the following: (*i*) TCs in the enteric wall (colon) have an endocytic (phagocytic-like) property, (*ii*) phTCs conserve the shape of TCs and maintain their typical distribution in the enteric wall, (*iii*) scarce ICCs have an endocytic property, (*iv*) CD34+ stromal cells in other locations also have the phagocytic-like capacity to uptake endogenous or exogenous particles, which may be an exponent of an endocytic general function of TCs and (*v*) there are evident differences between phTCs and macrophages, including cell morphology, distribution and size of the particles, and location and behaviour of these cells. Further studies in different locations and by means of other procedures are required, above all to investigate whether this ability to internalize small particles is generalized to all TCs and whether it is related to the cellular bidirectional cooperative circle of informative exchange and intercellular interaction.
